# Nociceptive pain assessed by the PainDETECT questionnaire may predict response to opioid treatment for chronic low back pain^☆^

**DOI:** 10.1016/j.heliyon.2024.e25834

**Published:** 2024-02-06

**Authors:** Stone Sima, Samuel Lapkin, Zachary Gan, Ashish D. Diwan

**Affiliations:** a Spine Labs, St George and Sutherland Clinical School, University of New South Wales, New South Wales, Australia; b Faculty of Health, Southern Cross University, Bilinga, Queensland, Australia; c Spine Service, Department of Orthopaedic Surgery, St George and Sutherland Clinical School, University of New South Wales, New South Wales, Australia

**Keywords:** Low back pain, PainDETECT, Neuropathic pain, Nociceptive pain, Pharmacological management, Medication efficacy, Opioids

## Abstract

**Introduction:**

The pharmacological management of chronic low back pain (LBP) is complex. The World Health Organisation recommends a laddered approach to pain medication usage. The PainDETECT questionnaire distinguishes between neuropathic pain (NeP), nociceptive pain (NoP), and ambiguous pain. By elucidating the difference in medication efficacy between these groups, clinicians can provide a tailored treatment plan to manage patient's pain. This study aimed to investigate the relationship between pharmacological treatments, pain categorizations, and medication efficacy as reported by patients.

**Methods:**

A secondary retrospective analysis of a prospectively collected database was conducted involving 318 consecutively recruited patients, aged 18 years and above, who completed PainDETECT, medication history and patient reported medication efficacy questionnaires. Medication history was categorized into four lines of treatment: first line (paracetamol ± non-prescribed anti-inflammatories), second line (prescribed anti-inflammatories), third line (anticonvulsants/neuromodulators) and fourth line (opioids). Medication efficacy was measured using a three-point Likert scale: effective (+2), somewhat effective (+1), no effect (0).

**Findings:**

The study included 120, 50, 54 and 94 patients on first line, second line, third line and fourth line treatment, respectively. The NeP group had higher mean numerical rating scale (NRS) compared to NoP group in all four lines of treatment (8.10 ± 1.59 vs. 5.47± 2.27, p < 0.001, 8.64± 1.43 vs. 5.52± 1.86, p < 0.001, 8.00± 1.07 vs. 6.37± 2.39, p < 0.01, and 8.05± 1.73 vs. 7.2± 1.29, p < 0.05). When confounding for severity of LBP as measured by NRS, the distribution of medication efficacy significantly differed amongst the NeP, ambiguous and NoP groups in patients undergoing fourth line pharmacological treatment (r^2^ = 8.623, p < 0.05). The NoP group exhibited significantly higher medication efficacy compared to the NeP group (U = 14.038, p < 0.05). There was no significant difference in medication efficacy across the pain classifications for first, second- and third-line treatment.

**Interpretation:**

Opioids was the only line of treatment more effective in targeting NoP, as determined by the PainDETECT questionnaire, compared to NeP. This pioneering study illustrates the complex nature of pharmacological management for chronic LBP. It underscores the importance of tailoring pharmacological treatment plans to fit individual pain profiles and expectations instead of adopting a blanket approach to pain management.

Funding **Disclosures:** This work was supported by a University Postgraduate Award from The 10.13039/501100001773University of New South Wales to SS. Spine Labs is supported via unrestricted research grants to its institution by 10.13039/100004702Baxter Inc and Nuvasive Inc.

## 1 Introduction

Low back pain (LBP) is the most common indication for patients being referred to a tertiary spine clinic by a general practitioner [1]. Chronic LBP lasting more than 3 months is prevalent in 50% of LBP sufferers, as a result LBP leads to increased disability, lower quality of life and psychological distress [2]. It is hypothesized that LBP is either resultant from activation of nociceptors known as nociceptive pain (NoP), or neuropathic pain (NeP) [3]. NeP is defined by the International Association for the Study of Pain as ‘pain caused by a lesion or disease of the somatosensory nervous system [4]. The PainDETECT questionnaire (supplementary 1) is the only validated screening tool to discriminate between NeP and NoP on a 38 point scale, with 0–12 being negative of NeP, 13–18 being an ambiguous result and 19–38 being positive of NeP [5].

Conservative management of chronic LBP comprises primarily of pharmacotherapy. Paracetamol (acetaminophen) and non-steroid anti-inflammatory drugs (NSAIDs) have anti-pyrectic and analgesic actions. Studies have only shown NSAIDs and paracetamol to be effective in patients with chronic LBP without sciatica and NeP signs [[6], [7], [8]]. The positioning of neuromodulating agents and opioids, is heavily debated by national and international guidelines in treating chronic LBP. A review by Bates et al. demonstrated that most guidelines recommend a trial of neuromodulating agents for patients in whom other agents have not been effective before beginning traditional opioids [9]. Pregabalin and gabapentin are anticonvulsants with neuromodulating effects, however, there efficacy in treating neuropathic pain is highly controversial [10,11].

Opioids serve as a final conservative treatment method before surgical intervention is required. The rate of opioid prescription for the long term management of chronic LBP has steadily risen, however, the benefits and risks of these medications remains heavily debated. Apart from its analgesic properties, it is well known that opioids pose inherent health complications like drug tolerance and addiction [[12], [13], [14]]. Few studies have conducted subgroup analysis on chronic LBP patients to determine the efficacy of opioids on NoP and NeP. One meta-analysis found opioids were more effective than placebo for both NoP and NeP [15], on the other hand another study failed to find a significant difference in opioid efficacy when compared with placebo [16]. Finding potential predictors of response can reduce opioid associated risk by allowing more selective prescription of opioids and lower doses.

No study to date has conducted an analysis comparing the efficacy of a certain drug in targeting NeP compared to NoP type of pain in the context of the lower back. Previous published studies exploring the use of certain medications in NeP have yielded controversial conclusions. Therefore, it is interesting to ponder whether there is a difference in efficacy of medications administered to patients suffering from NeP versus NoP. By elucidating these differences clinicians can provide a tailored treatment plan to precisely manage each individual patient's pain condition with the potential to enhance patient outcomes. This inquiry touches upon the broader issue of pain classification, treatment mechanisms and distinctiveness of therapeutic responses. However, such information may be difficult to elucidate with RCT's and there may be a role to glean the information by a careful real-world observation of prospectively collected data. Hence, a comparison study was conducted to establish the difference in efficacy between certain medications in treating NeP compared to NoP within a spinal pain referral centre.

## 2 Materials and methods

### 2.1 Study design and patient population

The study was IRB approved for a secondary analysis of an ongoing prospectively collected database. Written approval for the use of an electronic format PainDETECT questionnaire was granted by the original developer for the purpose of this study [5]. The study was conducted as a retrospective cohort study of adult patients (over 18 years of age) suffering with chronic (over 6 months) low back and/or leg pain who presented to a tertiary spine clinic with completed PainDETECT questionnaires and answered two questions addressing the medications they are currently using and the efficacy represented by a 3-point Likert scale. Patients were excluded if they had a history of lumbar spinal injection or surgery prior to completing the PainDETECT form. Written informed consent was obtained from all patients to be included in the study. The data set was used to perform an analysis of the efficacy of certain medication in treating NoP and NeP pain.

### 2.2 Data collection

PainDETECT scores and demographic information encompassing patient sex age were extracted from the tertiary spine clinic's PainDETECT RedCap database. The severity of LBP was measured using the 0 to 10 numerical rating scale (NRS). PainDETECT score was trichotomized into the NoP, ambiguous and NeP pain groups as defined by the PainDETECT questionnaire. The evaluation of medication efficacy employed a 3-point Likert scale, which is treated as a non-interval ordinal parameter. In this context, each step up in the scale denoted a non-uniform increase in medication efficacy (Table 1). The patient reported medications were classified into four distinct categories (Table 2). To maintain study coherence, an adapted pain escalation treatment protocol was implemented for patients utilizing multiple medications. It is noteworthy that only the highest line medication within this hierarchy was recorded [9]. Medications not contained within the four lines of treatment were also extracted.

**Table 1:** Patient initial intake assessment. Patient reported medication efficacy and corresponding Likert scale ratings.

Likert Scale Score	Medication Efficacy Question
	Does the medication:
+2	Relieve your symptoms a great deal
+1	Relieve your symptoms somewhat
0	Has/have no effect

**Table 2:** Categorization and corresponding line of medication patients reported to be taking for their current spine problem.

Line of treatment	Medication category
1st line	Panadol/Paracetamol/Acetaminophen +/− non-prescribed anti-inflammatories (ibuprofen, Diclofenac, Naproxen, Aleve)
2nd line	Prescribed anti-inflammatories (Mobic, Celebrex)
3rd line	Neuromodulating agents (Lyrica, Pregablin)
4th line	Opioids (Endone, Palexia, Tramadol, Morphine, Oxycodone, Targin)

### 2.3 Statistical analysis

Numerical variables were presented as mean ± standard deviation (SD). Categorical variables were summarized using counts (n) and percentages (%). Kruskal-Wallis H test was used to analyse the difference in medication efficacy for more than two groups. A post-hoc Mann-Whitney *U* test was used to analyse the difference in medication efficacy between paired groups. Analysis of covariance (ANCOVA) was used to analyse the difference in medication efficacy between NeP, NoP and ambiguous LBP classifications when confounded for severity of LBP. One way analysis of variance (ANOVA) was used to analyse the difference in continuous variables between more than two groups. Statistical analyses were conducted using the commercially available software SPSS (version 20, IBM Corporation, New York, USA). The level of statistical significance was set at 5% (p = 0.05).

## 3 Results

### 3.1 Demographics

**Fig. 1:**
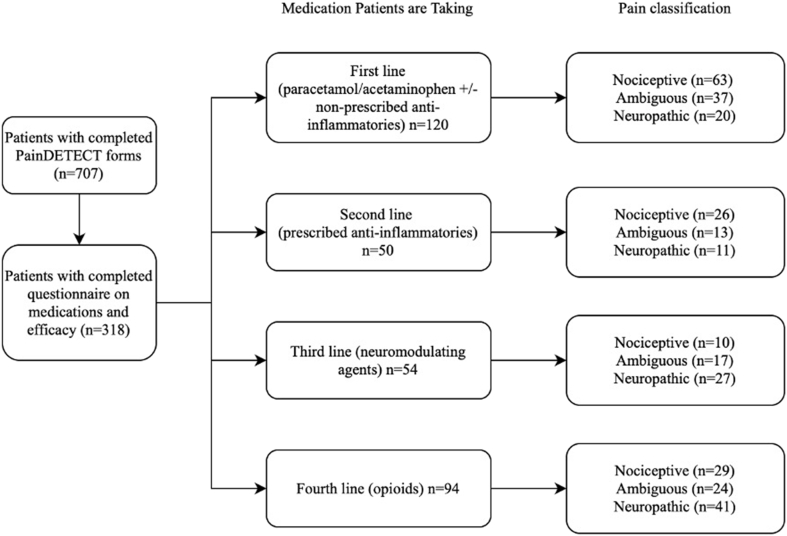
Flowchart depicting the inclusion of participants in the study. Flowchart representing the process of patient inclusion and exclusion into the study with the specific data on the number of patients included/excluded at each step. It also shows how the patients in the study were divided into the four lines of treatment and subsequently divided into nociceptive, ambiguous, and neuropathic categories as described by the PainDETECT questionnaire.

A flowchart depicting patient inclusion, exclusion, and separation into different medication and pain classification groups is shown in Fig. 1. Table 3 shows the demographic and clinical information of included patients. Out of the 318 patients included in the study, 120 had first, 50 had second, 54 had third and 94 had fourth line treatment for chronic LBP lasting more than 6 months. The neuropathic group had highest mean NRS compared to ambiguous and nociceptive group for first (8.10 ± 1.59 vs. 7.09± 1.63 vs. 5.47± 2.27, p < 0.001), second (8.64± 1.43 vs. 7.75± 1.06 vs. 5.52± 1.86, p < 0.001) and third line (8.00± 1.07 vs. 6.94± 1.39 vs. 6.37± 2.39, p < 0.01) treatment. In the fourth line treatment the neuropathic group had highest mean NRS compared to nociceptive and ambiguous group (8.05± 1.73 vs. 7.2± 1.29 vs. 6.96± 1.57, p < 0.05). No significant difference was also observed in the number of patients taking other medications between the three pain classification groups.

**Table 3:** Patient demographics and clinical data categorized into different treatment groups and pain classifications.

Parameter	NoP	Ambiguous	NeP	P-value	Total
First line treatment					
Age (mean ± SD)	54.78 ± 20.09	55.11 ± 18.81	50.35 ± 14.54	0.616	54.14 ± 18.82
Sex (F/M)	35/28	17/20	12/8	0.524	64/56
NRS (mean ± SD)	5.47 ± 2.27	7.09 ± 1.63	8.10 ± 1.59	**<0.001**	6.42 ± 2.24
**Second line treatment**					
Age (mean ± SD)	55.00 ± 18.75	65.31 ± 15.10	52.36 ± 14.48	0.130	57.10 ± 17.41
Sex (F/M)	11/15	7/6	6/5	0.703	24/26
NRS (mean ± SD)	5.52 ± 1.86	7.75 ± 1.06	8.64 ± 1.43	**<0.001**	6.85 ± 2.08
**Third line treatment**					
Age (mean ± SD)	60.70 ± 17.71	52.24 ± 15.97	58.44 ± 15.53	0.334	56.91 ± 16.11
Sex (F/M)	5/5	10/7	13/14	0/782	28/26
NRS (mean ± SD)	6.37 ± 2.39	6.94 ± 1.39	8.00 ± 1.07	**<0.01**	7.40 ± 1.55
**Fourth line treatment**					
Age (mean ± SD)	65.38 ± 16.26	62.83 ± 12.49	58.24 ± 15.35	0.145	61.62 ± 15.35
Sex (F/M)	16/13	14/10	25/16	0.889	55/39
NRS (mean ± SD)	7.2 ± 1.29	6.96 ± 1.57	8.05 ± 1.73	**<0.05**	7.5 ± 1.63
**Other Medication**					
Muscle relaxants (Y/N)	6/75	5/56	11/60	0.213	22/191
Benzodiazepines (Y/N)	4/77	2/59	7/64	0.248	13/200

Pearson chi-square test and analysis of variance (ANOVA) test were used to assess association and compared differences between the nociceptive, ambiguous, and neuropathic group as defined by the PainDETECT questionnaire. M: male, F: female, %: percentage, SD: standard deviation, Y: yes, N: no.

### 3.2 Efficacy of medication for NeP versus NoP

Kruskal-Wallis test showed that medication efficacy rank was significantly different across the trichotomized pain classifications of NoP, ambiguous and NeP for patients in the fourth line treatment group (H(2) = 8.623, p < 0.05). Post hoc Mann-Whitney test using a Bonferroni correction for multiple tests showed a significantly higher medication efficacy in the nociceptive group compared to the neuropathic group (U = 14.038, p < 0.05). There was no significant difference in medication efficacy rank for the first, second and third line treatment groups (Table 4).

**Table 4:** Difference in medication efficacy between pain classification groups across the different lines of treatment.

Kruskal Wallis Test	Test statistic	P-value
First line treatment	2.727	0.256
Second line treatment	2.228	0.328
Third line treatment	0.206	0.902
Fourth line treatment	8.623	**<0.05**
NeP-Ambiguous	12.205	0.082
NeP-NoP	14.038	**<0.05**
Ambiguous-Nop	1.833	0.757

Kruskal Wallis test with a Mann-Whitney U post hoc analysis was used to compare the differences in medication efficacy between pain classification in different lines of treatment. NoP: nociceptive pian, NeP: neuropathic pain.

### 3.3 Efficacy of medication when confounded for severity of LBP

ANCOVA test showed that when confounded for NRS, medication efficacy was significantly different across the trichotomized pain classifications of NoP, ambiguous and NeP for patients in the fourth line treatment group (r^2^ = 0.167, p < 0.05). The NoP and ambiguous group had higher mean medication efficacy compared to NeP group. There was no significant difference in medication efficacy for first, second and third line treatment groups when confounded for NRS (Table 5).

**Table 5:** Difference in medication efficacy between pain classification groups across the different lines of treatment when confounded for numerical rating scale.

ANCOVA	Adjusted NRS	R squared	P-value
First line treatment	6,46	0.104	0.416
Second line treatment	6.84	0.088	0.583
Third line treatment	7.40	0.013	0.887
Fourth line treatment	7.48	0.167	**P<0.05**
Pain Classification		Mean Medication Efficacy	
NoP		0.892	
Ambiguous		0.869	
NeP		0.650	

Analysis of covariance (ANCOVA) was used to compare the differences in medication efficacy between pain classification in different lines of treatment when confounded for numerical rating scale (NRS). NoP: nociceptive pain, NeP: neuropathic pain.

## 4 Discussion

This study was a retrospective cohort analysis based on data from an ongoing prospectively collected database. The aim of the analysis was the examine the association between the different classifications of pain (NeP, ambiguous and NoP) as defined by the PainDETECT questionnaire with efficacy of fourth different lines of pharmacological treatment. Of the 318 patients included 120 were receiving first line treatment consisting of paracetamol ± non-prescribed anti-inflammatories, 50 were receiving second line treatment consisting of prescribed anti-inflammatories, 54 were receiving third line treatment consisting of neuromodulating agents and 94 were receiving fourth line treatment consisting of opioids. In all four lines of treatment the patients experiencing NeP had the highest NRS, which corresponds to previous literature finding NeP patients to experience higher levels of pain, disability, and reduced quality of life [17].

Paracetamol/acetaminophen provides analgesic and antipyretic effects; however, it lacks anti-inflammatory activity [18]. Consequently, it only offers pain relief without addressing the causes of NeP, which involves neuronal compression and inflammation. The efficacy of prescribed anti-inflammatories, namely COX-2 selective inhibitors, in treating NeP hyper-inflammation remains unexplored. Existing research only demonstrated improved patient-reported outcomes for NoP cases [[6], [7], [8]]. This study demonstrated equivalent efficacy in patients taking prescribed anti-inflammatories for NeP and NoP. Although both have inflammatory pathologies, NoP stems from acute inflammation of the sinuvertebral nerve, whilst NeP is linked to chronic elevation of inflammatory mediators in the intervertebral disk (IVD) [19,20]. Consequently, the use of COX-2 inhibitors for NeP is limited as it has relatively poor analgesic efficacy and is associated with an increase in adverse reactions.

The role of neuromodulating agents in treating NeP is heavily debated. Pregabalin and gabapentin, known as gabapentanoids, are the most commonly prescribed in tertiary spinal clinics. They modulate pathologically enhanced neurotransmission in the central terminals of primary afferent neurons, therefore targeting the neuropathic component of chronic LBP [21]. While evidence supporting the efficacy of these drugs in NeP treatment is limited, their prescription rates for chronic LBP have increased substantially in recent years [22]. Studies indicate significant improvement in patient-reported outcome scores with pregabalin or gabapentin compared to baseline and placebos [23,24]. Contrarily, our study interestingly found that neuromodulating agents have no increase in effectiveness in treating NeP compared to NoP. A meta-analysis of nine trials comparing neuromodulating agents to placebos corroborated this, asserting their ineffectiveness in addressing low back pain or lumbar radicular NeP. Moreover, it revealed robust evidence linking gabapentanoid use to an elevated risk of adverse events [25], encompassing somnolence, dizziness, weight gain, and peripheral oedema [26]. Given the relatively poor benefit to risk ratio for treating NeP with gabapentanoids, it should only be considered as an option for patients who do not respond to other analgesic agents and can tolerate them.

The use of opioids in patients with chronic LBP is very controversial. Although studies have shown opioids to be effective in treating chronic LBP, no subgroup analysis of patients only experiencing NoP or NeP has been performed [27]. Consequently, the effectiveness of opioids for chronic LBP patients experiencing NoP or NeP without a definitive diagnosis remains uncertain. Pharmacologically, opioids bind to G-protein coupled opioids receptors on the descending inhibitory pathway to modulate the generation and transmission of pain impulses [28]. Consequently, opioids can only offer analgesic effects and do not target the root causes of NeP, namely neuronal compression and chronic hyperinflammation of the degenerated IVD. This was supported by our study which found when confounded for NRS pain level, patients taking opioids in the NoP group reported higher medication efficacy compared to the NeP group. This result was also corroborated by Hale et al. who demonstrated a significant reduction in pain intensity in patients taking opioids for chronic LBP without radiculopathy compared to placebo [29]. However, apprehensions concerning the development of analgesic tolerance, opioid dependency, and addiction among patients constrain the practicality of widespread opioid prescription [30,31]. Despite these constraints, the PainDETECT questionnaire could serve as a tool for opioids to be employed safely and effectively for patients with chronic NoP-associated LBP if factors contributing to inadequate response are identified pre-emptively through comprehensive medical and psychosocial histories. Ultimately, it would be premature to outright dismiss opioids as a treatment avenue for chronic NoP-related LBP. Future investigations should scrutinize potential markers to predict response to opioid treatment. This approach would facilitate targeted interventions preceding opioid therapy, aimed at transforming non-responsive patients into individuals bearing these indicative biomarkers.

The traditional pharmacological approach to chronic LBP has a notable challenge: a hasty, one-size-fits-all pain management ladder strategy that overlooks potential benefits versus heightened adverse effects. This could stem from prescribing pain medications without prior comprehension of the pain biomarkers underlying NoP and NeP. Interestingly, this study found medication that target NeP to have no increase in efficacy compared to NoP, positing that patients suffering NeP may only demonstrate significant clinical improvement when managed via a surgical approach. Surprisingly, a comprehensive pain profile combining patient biomarkers and microbiome for diagnosis, treatment, and enhanced pathophysiology understanding is still absent. This profile could help identify markers predicting responsiveness to different treatment tiers.

The study's findings were influenced by several limitations. Primarily, the study had a retrospective design and assessed medication efficacy solely based on patient-reported Likert scale scores. Moreover, the study didn't stratify the population to focus exclusively on patients receiving a single medication type, making it challenging to isolate the effectiveness of individual medications across different pain categories. However, in the real-world patients try a range of medications based on numerous factors. To delve deeper into the pharmacological management of NeP, a prospective study is necessary. Such a study should analyse pre- and post-treatment outcomes while correlating them with specific biological markers in chronic LBP patients. Such an approach promises valuable insights for optimizing pain management and enabling tailored treatments, departing from the conventional uniform pain ladder strategy.

## 5 Conclusion

NeP had the highest NRS in all four lines of treatment compared to the NoP group.

Patients with NoP in the fourth line treatment group (opioids), when confounded for the same NRS, reported a higher medication efficacy compared to patients with NeP. No significant difference in medication efficacy was recorded between NeP and NoP in the first three lines of treatment. These results emphasise the multifactorial and complex nature of pharmacological management for chronic LBP. It underscores the importance of tailoring pharmacological plans to fit individual pain profiles instead of adopting a blanket ladder approach to pain management. It also demonstrates that the PainDETECT questionnaire can potentially be utilised as a screening tool to predict opioid response in patient suffering with chronic LBP. Future research should continue to explore the role of biological markers in patients who respond well to pharmacological management compared to those who do not, in order to build patient profiles that respond to each line of treatment.

## Data availability

The data that support the findings of this study are available from the corresponding author, SL, upon reasonable request. The data are not publicly available since the study participants did not provide consent for the public sharing of their data.

## Ethics statement

This study was approved by the Human Research Ethics Committee of the University of Wollongong (HREC No.2020/329) for the retrospective collection of anonymized patient's initial intake forms including medication details and PainDETECT data from digital archives. The study complies with all regulations set by the ethics committee of the University of Wollongong. Written consent was provided by all patients prior to enrolling into the study.

## Funding

This work was supported by a University Postgraduate Award from The 10.13039/501100001773University of New South Wales to SS. All authors declare that they have no known competing financial interests or personal relationships that could have appeared to influence the work reported in this paper. Spine Labs is supported via unrestricted research grants to its institution by 10.13039/100004702Baxter Inc and Nuvasive Inc.

## CRediT authorship contribution statement

**Stone Sima:** Writing – review & editing, Writing – original draft, Visualization, Validation, Supervision, Methodology, Investigation, Formal analysis, Data curation, Conceptualization. **Samuel Lapkin:** Writing – review & editing, Visualization, Supervision, Resources, Project administration, Methodology, Investigation, Conceptualization. **Zachary Gan:** Writing – review & editing, Writing – original draft, Validation, Investigation, Formal analysis, Data curation. **Ashish D. Diwan:** Writing – review & editing, Visualization, Validation, Supervision, Resources, Project administration, Investigation, Funding acquisition, Conceptualization.

## Declaration of competing interest

The authors declare that they have no known competing financial interests or personal relationships that could have appeared to influence the work reported in this paper.
