# Supplementary figures and images for: Nociceptive pain assessed by the PainDETECT questionnaire may predict response to opioid treatment for chronic low back pain
Source: Heliyon. 2024 Feb 6;10(3):e25834. doi: 10.1016/j.heliyon.2024.e25834 (PMC10865323; doi:10.1016/j.heliyon.2024.e25834)

**Supplementary 1**

**
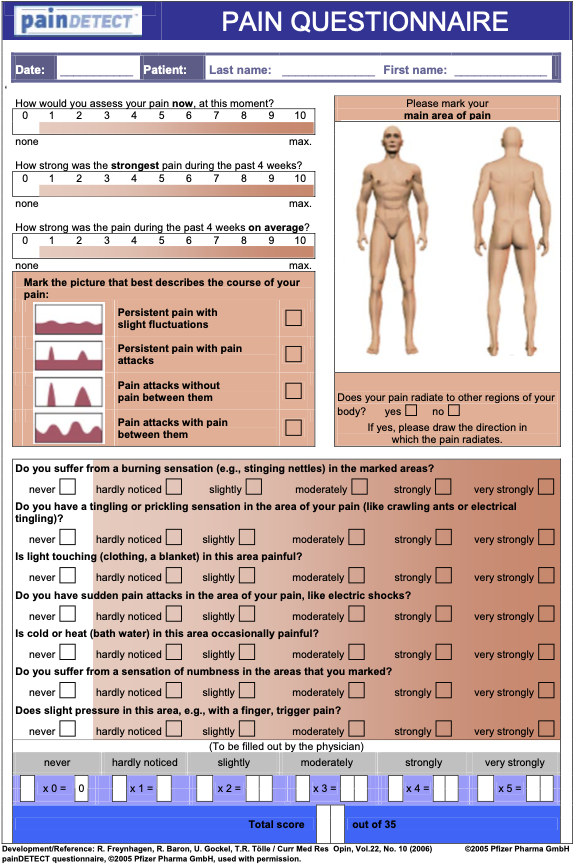
**

**
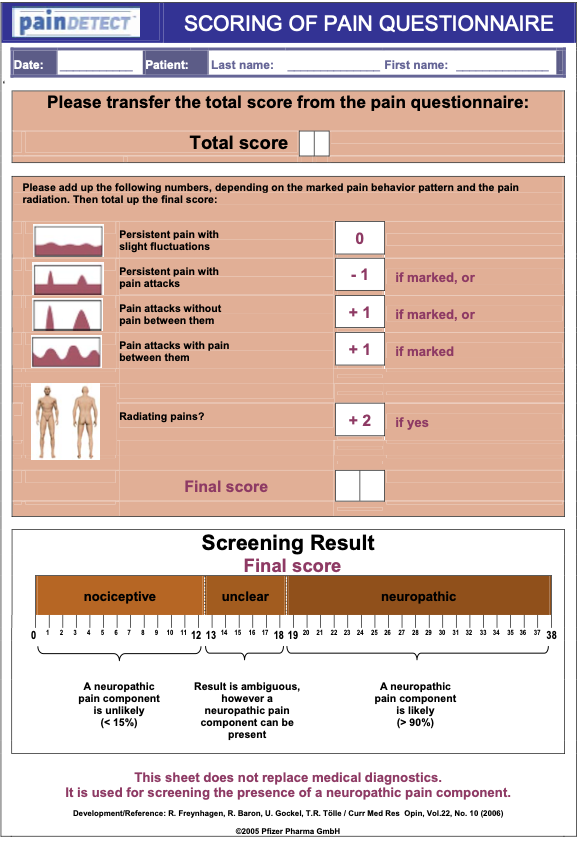
**

Note: The final/total score of the PainDETECT questionnaire is 38.

Supplement: Multimedia component 1 [file mmc1.docx]
